# Effect of somatic cell count and mastitis pathogens on milk composition in Gyr cows

**DOI:** 10.1186/1746-6148-9-67

**Published:** 2013-04-08

**Authors:** Carolina Barbosa Malek dos Reis, Juliana Regina Barreiro, Lucinéia Mestieri, Marco Aurélio de Felício Porcionato, Marcos Veiga dos Santos

**Affiliations:** 1 Department of Nutrition and Animal Production, School of Veterinary Medicine and Animal Sciences, University of São Paulo, Pirassununga, Brazil

**Keywords:** Somatic cell count, Gyr cows, Mastitis, Milk composition

## Abstract

**Background:**

Gyr cows are well adapted to tropical conditions, resistant to some tropical diseases and have satisfactory milk production. However, Gyr dairy herds have a high prevalence of subclinical mastitis, which negatively affects their milk yield and composition. The objectives of this study were (i) to evaluate the effects of seasonality, mammary quarter location (rear x front), mastitis-causing pathogen species, and somatic cell count (SCC) on milk composition in Gyr cows with mammary quarters as the experimental units and (ii) to evaluate the effects of seasonality and somatic cell count (SCC) on milk composition in Gyr cows with cows as the experimental units. A total of 221 lactating Gyr cows from three commercial dairy farms were selected for this study. Individual foremilk quarter samples and composite milk samples were collected once a month over one year from all lactating cows for analysis of SCC, milk composition, and bacteriological culture.

**Results:**

Subclinical mastitis reduced lactose, nonfat solids and total solids content, but no difference was found in the protein and fat content between infected and uninfected quarters. Seasonality influenced milk composition both in mammary quarters and composite milk samples. Nevertheless, there was no effect of mammary quarter position on milk composition. Mastitis-causing pathogens affected protein, lactose, nonfat solids, and total solids content, but not milk fat content. Somatic cell count levels affected milk composition in both mammary quarters and composite samples of milk.

**Conclusions:**

Intramammary infections in Gyr cows alter milk composition; however, the degree of change depends on the mastitis-causing pathogen. Somatic cell count is negatively associated with reduced lactose and nonfat solids content in milk. Seasonality significantly affects milk composition, in which the concentration of lactose, fat, protein, nonfat solids and total solids differs between dry and wet seasons in Gyr cows.

## Background

Milk composition and microbiological characteristics are important factors for the dairy farmer (raw milk quality), dairy industry (technological process and quality of dairy products), and consumer (nutritional quality and safety). Milk composition varies according to factors such as breed, age, mammary gland health, lactation stage, nutritional management, and season [1]. Nutritional management and modifications in diet composition are responsible for changes in milk protein and fat concentration. Dry matter intake, available fiber, and the diet energy:protein ratio are considered some of the main factors affecting the fat and protein content of milk [2].

Mastitis is one of the most common diseases in dairy cows and results in great economic losses in the dairy industry [3]. Mastitis is defined as a mammary gland inflammation that is generally caused by bacterial infections [4]. This disease negatively affects the physical-chemical characteristics, composition, and yield of milk [5]. These alterations are attributed to changes in vascular permeability due to the inflammatory process and the damage of epithelial cells that are responsible for the synthesis of milk components as well as changes in the enzymatic action of somatic cells or microorganisms in the infected mammary gland [6].

It is essential to monitor intramammary infections (IMI) in dairy cows in order to maintain milk quality and herd health. Several methods for diagnosing IMI are available, and the bacteriologic culture of milk samples is considered the standard method [7], but it is generally expensive and time consuming for routine screening. Individual somatic cell count (SCC) is currently used to screen for IMI status at the herd and cow levels because it is extensively available to dairy farmers and is less expensive than microbiological culture [8]. Increased SCC is associated with reductions in casein, milk fat, and lactose; increased enzymatic activity; and reduced quality and yield of dairy products [9].

Gyr cows represent an important percentage of Brazilian dairy cattle herds primarily because of their high adaptability to tropical climates and dairy production systems [10,11]. In Brazil, Gyr and crossbred cows (Gyr x Holstein) compose the majority of dairy herds. Gyr cows have been extensively used in tropical conditions because they are considered to be well adapted to this environment; they are also resistant to some tropical diseases and have satisfactory productive performance [11,12]. A recent study showed that Gyr dairy herds have a high prevalence of subclinical mastitis, which may negatively affect milk yield and composition [13]. However, the influence of mastitis-causing pathogens and SCC on milk composition in Gyr cows has not yet been determined.

The objectives of this study were (i) to evaluate the effect of seasonality, mammary quarter location (rear x front), mastitis-causing pathogen species, and somatic cell count (SCC) on milk composition in Gyr cows with mammary quarters as the experimental units and (ii) to evaluate the effect of seasonality and somatic cell count (SCC) on milk composition in Gyr cows with cows as the experimental units.

## Results

Milk composition results were obtained from milk samples from 3203 quarters (Data set 1) from 221 cows in three herds. The overall means were as follows: 4.48% (± 0.01) lactose, 3.67% (± 0.02) protein, 3.01% (± 0.04) fat, 9.13% (± 0.02) nonfat solids, and 12.15% (± 0.05) total solids contents in all composite samples (infected and uninfected).

The final mixed-model linear regression results of the effect of mastitis-causing pathogens and SCC on milk composition at the quarter level are shown in Table 1. The effect of IMI on milk composition was dependent on isolated mastitis-causing pathogens. For example, intramammary infection caused by all of the studied mastitis-causing pathogens reduced the total solids content of milk, with estimated reduction ranging from 0.16% ± 0.06 (*Corynebacterium* spp.) to 0.35% ± 0.11 (*Streptococcus sp.*). Compared with uninfected quarters (9.29%), nonfat solids content was also negatively affected by IMI caused by *Staphylococcus aureus,* coagulase negative staphylococci (CNS) and *Streptococcus sp.,* but not by *Corynebacterium* spp. Intramammary infections caused by CNS, *Streptococcus sp.*, and *Corynebacterium* spp. reduced milk fat content. Uninfected mammary quarters had estimated lactose content of 4.66%, but this value was reduced by IMI caused by *Corynebacterium* spp. (−0.08% ± 0.02). Intramammary infections caused by *S. aureus,* CNS and *Streptococcus sp.* resulted in no significant change in lactose content. Milk protein content was reduced by IMI caused by *S. aureus* (−0.04% ± 0.02) but was increased by IMI caused by *Corynebacterium* spp. (0.05% ± 0.02). No significant effect of IMI caused by CNS and *Streptococcus sp.* was observed for milk protein content.

**Table 1:** **Final mixed linear regression models of the effect of seasonality, mastitis-causing pathogens and somatic cell count on milk composition at the mammary quarter level in Gyr cows**^**1**^

	**Lactose, %**			**Protein, %**			**Fat, %**			**Nonfat solids, %**			**Total solids, %**		
**Fixed effects**	**β**	**SE**	**P-*value***	**β**	**SE**	**P-*value***	**β**	**SE**	**P-*value***	**β**	**SE**	**P-*value***	**β**	**SE**	**P-*value***
Intercept	4.660	0.081		3.662	0.087		1.689	0.335		9.295	0.098		11.004	0.427	
Seasonality^2^															
Dry	Ref.	…	…	Ref.	…	…	Ref.	…	…	Ref.	…	…	Ref.	…	…
Rainy	0.105	0.015	<.0001	−0.322	0.012	<.0001	−0.208	0.039	<.0001	−0.222	0.018	<.0001	−0.437	0.044	<.0001
IMI^3^															
Negative	Ref.	…	…	Ref.	…	…	Ref.	…	…	Ref.	…	…	Ref.	…	…
* S. aureus*	−0.041	0.024	0.089	−0.044	0.019	0.023	−0.082	0.062	0.183	−0.091	0.029	0.002	−0.191	0.071	0.007
CNS^4^	−0.023	0.022	0.294	−0.033	0.018	0.062	−0.175	0.057	0.002	−0.063	0.027	0.017	−0.241	0.065	0.0002
CORY^5^	−0.082	0.021	0.0002	0.052	0.017	0.003	−0.138	0.055	0.012	−0.015	0.025	0.554	−0.161	0.063	0.010
STREP^5^	−0.062	0.037	0.092	−0.031	0.029	0.297	−0.232	0.095	0.014	−0.099	0.044	0.023	−0.346	0.107	0.001
SCC^6^	−0.0002	0.00004	<.0001	0.00005	0.00004	<.0001	0.0002	0.00001	<.0001	−0.0002	0.00005	<.0001	0.00005	0.00002	<.0001

^1^ A total of 3,203 observations were used in this analysis. ^2^Seasonality (1 = rainy season, October to March; 0 = dry season, April to September), ^3^ IMI: intramammary infection; ^4^CNS: coagulase negative staphylococci, ^5^ CORY: *Corynebacterium* spp., ^6^ STREP: *Streptococcus* sp. (*S. uberis, S. agalactiae, S. dysgalactiae*), ^6^ SCC: somatic cell count (x 1000 cells/mL), β: estimated coefficients, SE: standard error for the coefficient, Ref.: reference.

Somatic cell count negatively affected the lactose and nonfat solids content of milk at the quarter level in Gyr cows. The mixed linear regression model estimated that each increase in SCC of 100,000 cells/mL reduced the lactose and nonfat solids content by 0.02%. However, SCC increased the protein, fat and total solids content. No effect of mammary quarter location on milk composition was detected between the front and rear mammary quarters (P > 0.05). For the mammary quarter samples, seasonality significantly influenced milk composition (P < 0.001). The levels of protein, fat, nonfat solids, and total solids were higher (3.77%, 1.98%, 9.22%, and 11.21%, respectively) and lactose content was lower (4.45%) during the dry season than during the wet season (3.43%. 1.68%, 8.98%, 10.67%, and 4.58%, respectively).

The final mixed-model linear regression results of the effect of seasonality and SCC on milk composition of composite samples are shown in Table 2. For the composite milk samples, SCC levels also influenced milk composition. Lactose and nonfat solids contents were negatively affected by SCC. Using a mixed linear regression model, it was estimated that each increase in SCC of 100,000 cells/mL reduced lactose and nonfat solids content by 0.02. Seasonality affected milk composition in the composite samples. Protein, fat, nonfat solids, total solids contents were higher (3.84%, 3.35%, 9.27%, and 12.63%, respectively) during the wet season than during the dry season (3.48%, 2.69%, 9.14%, and 11.83%, respectively). However, the opposite effect occurred with lactose; lower levels were observed during the wet season (4.43%) than the dry season (4.67%).

**Table 2:** **Final mixed linear regression models of the effect of mastitis-causing pathogens and somatic cell count on milk composition of composite milk samples in Gyr cows**^**1**^

	**Lactose, %**			**Protein, %**			**Fat, %**			**Nonfat solids, %**			**Total solids, %**		
**Fixed effects**	**β**	**SE**	**P-*value***	**β**	**SE**	**P-*value***	**β**	**SE**	**P-*value***	**β**	**SE**	**P-*value***	**β**	**SE**	**P-*value***
Intercept	4.755	0.053		3.432	0.061		2.477	0.348		9.168	0.073		11.655	0.387	
Seasonality^2^															
Dry	Ref.	…	…	Ref.	…	…	Ref.	…	…	Ref.	…	…	Ref.	…	…
Rainy	−0.243	0.028	<.0001	0.391	0.032	<.0001	0.616	0.096	<.0001	0.141	0.038	<.0003	0.746	0.106	<.0001
SCC^3^	−0.0002	0.00001	<.0001	0.00004	0.00001	<.002	0.0003	0.00004	<.0001	−0.0001	0.00002	<.0001	0.0001	0.00004	0.002

^1^ A total of 764 observations were used in this analysis. ^2^Seasonality (1 = rainy season, October to March; 0 = dry season, April to September), ^3^SCC: somatic cell count (x 1000 cells/mL), β: estimated coefficients, SE: standard error for the coefficient, Ref.: reference.

## Discussion

The results of the present study indicate that IMI negatively affects milk composition, although it has been shown that the degree of changes depends on the inflammatory response, the severity and amount of affected tissue in the mammary gland, and bacterial pathogenicity [14], which may explain the differences of the effect of mastitis-causing pathogens on milk composition found in this study. Milk composition is also affected by other factors such as age, lactation stage and nutritional practices [15]. However, these factors were not controlled in the present study.

Bansal et al. [15] determined that lactose content was higher (5.02%) in healthy quarters than in quarters with a SCC of >100,000 cells/ml and negative culture (non-specific mastitis) (4.71%), which are results similar to those found in the present study. Furthermore, these authors revealed a lower protein concentration (2.96%) in healthy quarter samples than in non-specific mastitis quarters (3.22%). However, in the present study, there was only a negative effect of IMI caused by *S. aureus* on protein content. In composite samples, Bansal et al. [15] reported a negative mastitis effect only with regard to lactose content, with a higher value (4.84%) for healthy samples than that for mastitis samples (4.61%).

It has been observed that milk samples from infected quarters have higher total protein and whey protein values, but lower casein content (proportion of casein relative to total protein) and lactose content when compared to milk samples from healthy quarters [16]. The increase in the permeability of blood-milk barrier during mastitis also results in an increased influx of serum proteins and enzymes from the blood, which may lead to an increase in proteolysis [16]. The findings reported by these authors showed an alteration in the protein composition in milk from affected quarters (SCC >100.000 cells/ml) when compared to milk from healthy quarters (SCC ≤100,000 cells/ml); the level of casein, including α-casein and β-casein, was lower in milk samples from affected quarters than milk samples from healthy quarters, whereas the level of whey protein was increased due to proteolysis by proteolytic enzymes or decreased casein synthesis. However, in this study, the protein content analysis was based on the total protein value. The percentages of casein and whey protein were not calculated, which may explain the overall non-significant effect of mastitis on protein content.

Some of the changes in milk composition are more marked than others; for example, lactose content is generally reduced as a result of mastitis due to decreased synthesis of this milk component [14]. For this reason, lactose concentration could be used as a mastitis indicator [14]. However, there are conflicting reports regarding the effect of mastitis on fat content. In the present study, IMI caused by CNS, *Streptococcus sp.*, and *Corynebacterium* spp. significantly reduced milk fat content, which differs from the results of Rogers et al. [17] that show no effect of mastitis on fat content. Kitchen [6] and Auldist et al. [18] reported a lower fat concentration in milk samples from cows with subclinical mastitis, whereas Mitchell et al. [19] reported an increased fat concentration in mastitis-positive samples. Bansal et al. [15] reported that for German cows, higher fat content (2.25%) was observed in foremilk quarter samples from non-specific mastitis samples than from healthy ones (1.43%).

It has also been reported that milk samples from Swedish cows with <100,000 cells/ml did not present a significant difference between their front and rear quarters with regard to protein content but presented lower fat and higher lactose contents (4.61% and 4.95%, respectively) in their rear quarters than in their front quarters (4.85% and 4.91%, respectively) [20]. In the present study, there was no effect of mammary quarter position on milk composition. Furthermore, an increase in protein, fat and total solids contents was observed as the SCC level increased. However, lactose and nonfat solids contents were affected in an opposite manner. A positive correlation between SCC and fat, and protein contents (r = 0.07 and r = 0.15, respectively) exists in composite samples from Holstein cows [5]. Several studies have demonstrated that the changes in milk composition caused by mastitis and high SCC (fat, protein, lactose, minerals, and enzyme concentration modifications) may be explained by epithelial cell damage (decrease in synthesis); an increase in vascular permeability with the passage of immunoglobulins, serum protein and minerals (sodium and chloride); and an increase in proteolytic activities [5,21,22].

In a previous study, Santos et al*.*[4] suggested that high SCC might cause changes in milk composition because milk samples with a SCC of >200,000 cells/ml undergo greater alterations in milk chemistry characteristics than samples with low SCC (≤200,000 cells/ml). Similarly, our results indicated that SCC linearly affects milk composition. In a study by Ogola et al*.*[23] with zebu cows, the lactose content and acid degree value were lower [4.3% and 0.60 meq of free fatty acid (FFA)/100 g fat, respectively] in samples with an SCC of >750.000 cells/ml than in samples with an SCC between 500,000 to 750,000 cells/ml (4.5% and 0.45 meq FFA/100 g fat, respectively), but no effect was observed for SCC on protein content [23]. Elevated SCC is accompanied by changes in milk composition, which may result in decreased cheese yield, increased rennet clotting time, reduction of the shelf-life of pasteurized milk, modifications in thermal stability, and others [22].

## Conclusion

Intramammary infections in Gyr cows alter milk composition. However, the degree of change depends on the mastitis-causing pathogen. Somatic cell count is negatively associated with reduced lactose and nonfat solids content in milk. Seasonality significantly affects milk composition, in which the lactose, fat, protein, nonfat solids and total solids concentration differs between the dry and wet seasons in Gyr cows.

## Methods

### Herd selection

A total of 221 lactating purebred Gyr cows from three commercial dairy farms located in the states of São Paulo and Minas Gerais, Brazil were selected on the basis of their proximity to our laboratory. Individual foremilk quarter samples and composite samples of milk were collected over 1 to 8 occasions from all lactating cows at the time of the farm visits, except from cows with clinical mastitis symptoms. Clinical mastitis was characterized by the presence of abnormal milk (flakes, clots, watery or discolored milk) or the presence of swollen or indurated mammary quarters before milking. Samples were collected monthly over one year.

All lactating cows were milked twice daily and had an average milk yield of 14 kg/day, average lactation length of eight months and parity number of 2.9 ± 1.8. Cows were managed on tropical pasture systems with free access to water and fed a concentrate supplement after milking according to milk yield and stage of lactation. Calving dates were assumed to occur at random over the course of the year because no information was available.

In the first herd, hand-milking was used, and there was no specific adoption of mastitis control practices at milking, although dry cow therapy and clinical mastitis treatment were employed. The second herd was milked using mechanic milking in a double-4 herringbone milking parlor. The milking routine included the following mastitis control practices: forestriping in a strip cup to check for clinical mastitis, pre-dipping, and drying teats with disposable towels. The second herd also adopted dry cow therapy and clinical mastitis treatment. In the third herd, cows were milked using machine milking in a double-6 tandem milking parlor with the same mastitis control program practices adopted by the second herd. In all three herds studied, calf suckling before milking was needed to stimulate milk let down. In all herds, post-dipping was not used because calves had access to udders for suckling at the end of each milking.

### Sampling and analysis

Sampling procedures were performed as described [13]. Briefly, before sampling, teat ends were immersed in iodine solution (0.5%), and after approximately 20 seconds, teats were dried with disposable towels. Each teat end was scrubbed with a cotton pledget saturated in ethyl alcohol (70%). The first streams of foremilk were discharged, and then approximately 10 mL of milk was aseptically collected for microbiological culture. Quarter milk samples were also collected and preserved with bronopol for SCC and milk composition analysis. Composite samples of milk were collected directly from the collection bucket immediately after milking and represent the whole milking of each animal. After collection, milk samples were stored at 4°C until bacteriological culture and SCC tests were performed. Milk composition and SCC was determined using the infrared and flow cytometry method using a Somacount 300® (Bentley Instruments, Chaska, MN) [24].

Bacteriological culture was performed according to NMC standards [25]. Briefly, from each sample, 0.01 mL of milk was plated on blood agar and incubated aerobically for 24 h or 48 h at 37°C. A mammary quarter was considered culture-positive when the growth of at least one colony was detected on the streaks. Samples yielding more than two different bacterial species were considered to be contaminated and removed from the statistical analysis (n = 31). Bacteria were identified based on colony morphology and Gram staining. For Gram-positive *cocci*, catalase tests with hydrogen peroxide (3%) were used to differentiate between catalase-positive *Staphylococci* and catalase-negative *cocci*. Coagulase tests were carried out using sterile rabbit plasma to distinguish *S. aureus* (coagulase-positive) from non-*aureus Staphylococci*, referred to as coagulase-negative S*taphylococci*. *Streptococci* were subdivided into esculin-positive *cocci* and esculin-negative *cocci* (*Strep. agalactiae* and *Strep. dysgalactiae*). CAMP-tests were used to differentiate *Strep. agalactiae* from *Strep. dysgalactiae.*

### Statistical analysis

Milk composition was modeled as a function of explanatory variables in mixed linear regression models using the PROC MIXED tool from SAS, version 9.2 (SAS Institute Inc., Cary, NC, USA). Two data sets were used; the first set (Data Set 1) consisted of individual foremilk quarter samples, and the second set (Data Set 2) consisted of composite milk samples. For the analysis of Data Set 1, the model included the following fixed effects: mammary quarter position, mastitis-causing pathogen and somatic cell count. Herd and cow within herd were included as random effects in all models. Backward elimination of independent variables was employed. The final linear mixed model for Data Set 1 was defined by the equation:

$$\begin{array}{l} {\text{Y}}_{\mathrm{ij}}=\beta_{0}+\beta_{1}{\mathrm{Seasonality}}_{\mathrm{ij}}+\beta_{2}{\mathrm{Pathogen}}_{\mathrm{ij}}\\ \;+\beta_{3}{\mathrm{SCC}}_{\mathrm{ij}}{}_{+}e_{\mathrm{ij}} \end{array}$$

where the subscripts *i* and *j c*orresponds to the ith herd and the jth cow, Y is the observed value (lactose, fat, protein, and nonfat solids contents), β_0_ is the intercept, β_1_ is the parameter for seasonality (entered as two dummy variables: 1 = rainy season, October to March; 0 = dry season, April to September), β_2_ is the parameter for mastitis-causing pathogen (entered as five dummy variables: culture-negative samples, *S. aureus*, coagulase-negative staphylococci, *Corynebacterium* spp., and others: *S. uberis, S. agalactiae, S. dysgalactiae*), β_3_ is the parameter for SCC (entered as continuous variable), and *e* is the error term.

For the analysis of Data Set 2 (composite milk samples), the final linear mixed model was defined by the equation:

$${\text{Y}}_{\mathrm{ij}}=\beta_{0}+\beta_{1}{\mathrm{Seasonality}}_{\mathrm{ij}}+\beta_{2}{\mathrm{SCC}}_{\mathrm{ij}}{}_{+}e_{\mathrm{ij}}$$

where the subscripts *i* and *j* corresponds to the ith herd and the jth cow, Y is the observed value (lactose, fat, protein, and nonfat solids contents), β_0_ is the intercept, β_1_ is the parameter for seasonality (entered as two dummy variables: 1 = rainy season, October to March; 0 = dry season, April to September), β_2_ is the parameter for SCC (entered as continuous variable), and *e* is the error term. For all statistical analyses, significance was considered at *P* ≤ 0.05.

## Competing interests

The authors declare that they have no competing interests.

## Authors’ contributions

CBMR was responsible for the milk sample collection, laboratory analysis and milk culture, and drafted the manuscript. JRB aided in the milk sample collection, laboratory analysis and milk culture, and drafted the manuscript. LM aided in the laboratory analysis and milk culture. MAFP participated in the milk sample collection, data interpretation, and revision of the manuscript. MVS participated in the design of the study, data interpretation and revision of the manuscript. All authors read and approved the final manuscript.
